# C-Reactive Protein, the Gliovascular Unit, and Alzheimer’s Disease

**DOI:** 10.7759/cureus.67969

**Published:** 2024-08-27

**Authors:** Mihaela Straistă, Mark Slevin

**Affiliations:** 1 General Medicine, The George Emil Palade University of Medicine, Pharmacy, Science, and Technology of Târgu Mureș, Târgu Mureș, ROU; 2 Center for Advanced Medical and Pharmaceutical Research, The George Emil Palade University of Medicine, Pharmacy, Science, and Technology of Târgu Mureș, Târgu Mureș, ROU

**Keywords:** monomer of crp, alzheimer's disease, neuroinflammation, gliovascular unit, mcrp, c-reactive proteins(crp)

## Abstract

Alzheimer's disease (AD) pathogenesis is conditioned by the presence of amyloid beta (Aβ) and neuroinflammation. The gliovascular unit (GVU) illustrates the relationship between the vascular components of the brain and glial cells, particularly astrocytes, which are seen as critical elements mainly affected in this disease. In AD patients, the impairment of the GVU is seen as blood-brain barrier breakdown, decreased clearance of Aβ, and chronic inflammatory status. C-reactive protein (CRP) and its monomeric form (mCRP) are associated with endothelial dysfunction and amyloid plaque instability, contributing to neuroinflammation and neurodegeneration. The interconnections between the GVU and the dissociated form of CRP were demonstrated by mCRP implication in vascular permeability that supports inflammation and extravasation of pro-inflammatory cytokines into the brain parenchyma. Astrocytic activation and endfeet function alterations can exacerbate the progression of AD by elevating pro-inflammatory agents and vascular amyloid accumulations. This review aims to emphasize the synergistic link between the GVU and monomers of CRP in the perpetuation of the inflammatory status, exacerbating neurodegeneration and neuroinflammation. Understanding their implication in AD can bring insights into novel therapeutic strategies to reduce AD progression.

## Introduction and background

Alzheimer’s disease (AD) represents a neurodegenerative disorder and has essential neuropathological characteristics: amyloid beta (Aβ) plaques and neurofibrillary tangles (NFT) [1]. Aβ accumulations can alter the neurovascular unit (NVU), leading to diminished cerebral blood flow (CBF) and blood-brain barrier (BBB) impairment. Along with vascular changes, various morphological and inflammatory response modifications occur at the level of neurons, glial cells, and cerebral blood vessels [2,3]. An early step in the development of dementia is NVU dysfunction and glial impairment. Takata F et al. [4] showed using primary cultures of rat brain that activated microglia can produce tumor necrosis factor-α (TNF-α), which triggers matrix metalloproteinase-9 (MMP-9) release and, via its proteolytic activity, might relate to pericyte loss and BBB damage. These findings highlight the strong interaction between vasculature and glial cells and emphasize the importance of the gliovascular unit (GVU) in AD.

C-reactive protein, particularly the monomeric form (mCRP), is claimed to be involved in neuroinflammation and NVU disruption. Slevin M et al. [5] showed that the inter-cellular gap became broader, and the vascular permeability increased in sprouts that emerged from mCRP-treated spheroids of rodent models.

Methods

This review aims to investigate the implication of glial cells in AD pathogenesis and their association with cerebral vasculature by examining the structure and function of the GVU. Furthermore, the interaction between GVU components and mCRP, concurrent with their involvement in neuroinflammation, development, and progression of AD is explored.

This narrative review includes sources provided by online databases such as Google Scholar, PubMed, and ScienceDirect. The following keywords were used in search engines: AD, GVU, mCRP, neuroinflammation, and C-reactive protein.

## Review

Gliovascular unit

**Figure 1:**
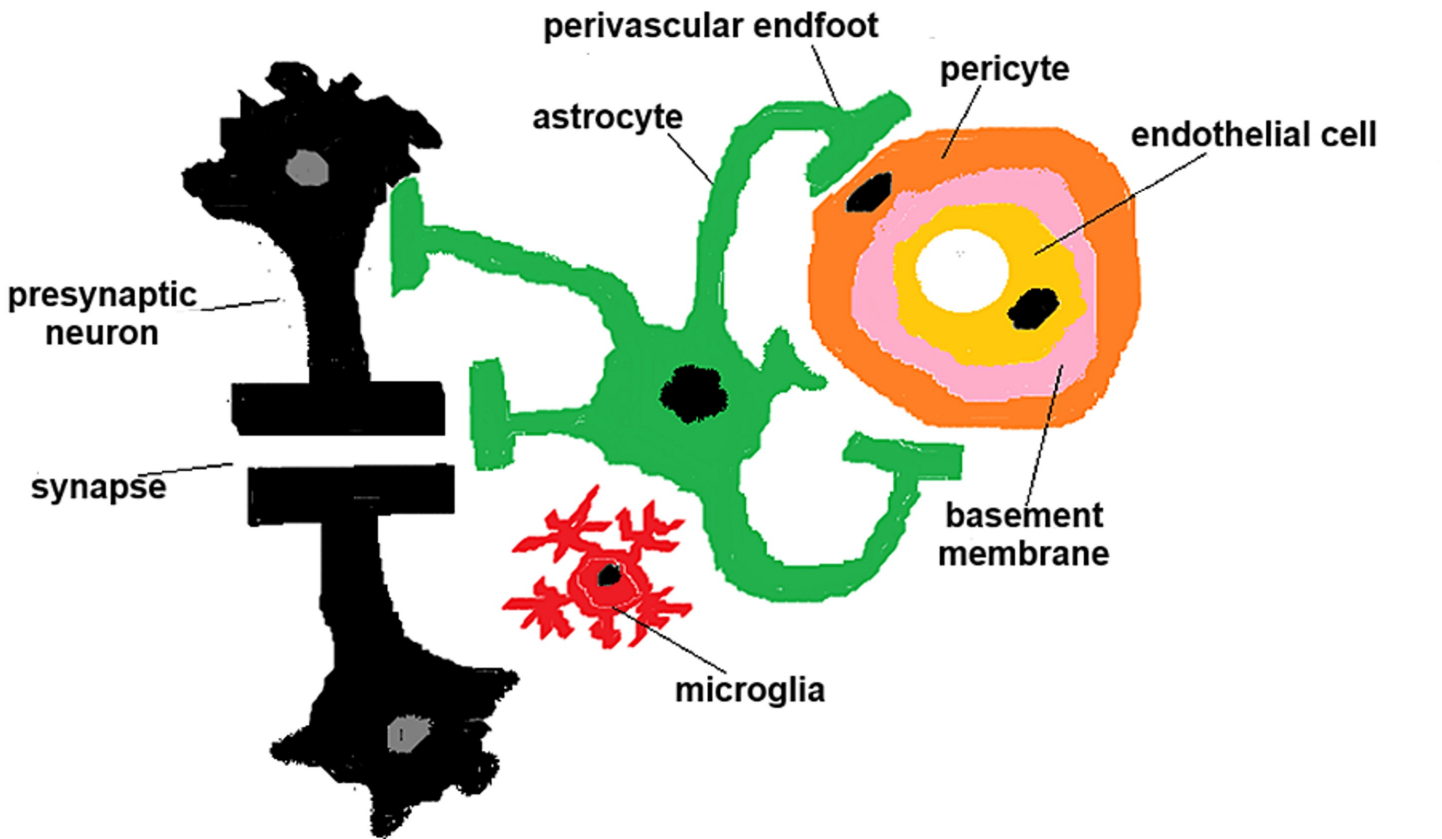
Gliovascular unit at the level of a brain capillary. It comprises glial cells (astrocytes, microglia) and vascular cells (endothelial cells, pericytes). Image Credit: Straistă Mihaela.

GVU implies the structural and functional synergy between the glial cells and cerebral vasculature. It exerts a pivotal role in blood flow regulation, BBB preservation, and metabolic sustenance (Figure 1) [6].

Astrocytes Activation

In response to brain injury, astrocytes proliferate and undergo specific changes, a process known as astrocytosis or astrogliosis. The activated astrocytes release signaling molecules and pro-inflammatory agents that include cytokines, namely transforming growth factor β (TGF-β), TNF-α, chemokines, prostaglandins (PG), nitric oxide (NO), and gliotransmitters [7].

Moreover, studies have shown that reactive astrocytes can enzymatically degrade Aβ peptides by cleaving them at a single or multiple sites. These enzymes are represented by endothelin-converting enzyme (ECE), neprilysin (NEP), insulin-degrading enzyme (IDE). Other mechanisms that assure Aβ clearance involve apolipoprotein J, alpha 2-macroglobulin (α2-M), and apolipoprotein E (ApoE) which alter the ability of forming insoluble aggregates by binding to Aβ species [8].

Astrocytes Endfeet: A Mediator in Vasculature Control

Astrocytes have a complex relationship with cerebral vasculature. The close association between these two entities is ensured by astrocytes’ end-feet, offering 70-100% covering of the vascular perimeter and providing isolation of vasculature from the brain parenchyma. These structures have multiple functions such as preserving BBB integrity, controlling blood flow, brain waste clearance, and nutrient uptake [9]. Aquaporin-4 (AQP4) is one of the generally studied endfeet proteins. It is involved in the regulation of water transport and osmotic balance [10].

AD Alterations in Endfeet Function

Astrocytes withstand critical phenotypical changes like the upregulation of endfoot gene expression of AQP4 and megalencephalic leukoencephalopathy with subcortical cysts 1 (MLC1). Studies based on human brains of AD patients and mouse models with amyloidopathy demonstrated an upregulation of AQP4 in the cortex. Moreover, along the progression of AD, a reduction of AQP4 polarization is noticed [10]. These changes are specifically remarked around vessels that present CAA. Vascular amyloid accumulations are formed between vascular mural cells and the astrocyte endfeet, leading to diminished vessel coverage and swollen presentation of the endfeet themselves. This physical spacing between EC and endfeet results in NVU impairment, with BBB disruption and neuronal nutrition deprivation [11]. In summary, the significance of astrocyte endfeet interaction with cerebral vasculature in AD patients is a topic of high interest that demands more investigation, due to their role in CNS (central nervous system) homeostasis and BBB function.

ApoE Polymorphism Seen as a Risk Factor for Late-Onset of AD

ApoE is considered one of the most consistently replicated risk factors for late-onset sporadic AD, and the ApoE4 isoform is associated with a greater risk. ApoE4 carriers are found to have a higher rate of pericyte degeneration and BBB dysfunction by restricting Aβ clearance and decreasing CBF [12]. Because of the BBB leakage conditioned by ApoE4 status, inflammatory elements such as CRP from the periphery can infiltrate into the brain and promote inflammation. The Framingham Heart Study offspring cohort revealed that chronically elevated levels of CRP can raise the risk of AD in ApoE4 positive patients [13]. Gan Q et al. [14] investigated the effects of mCRP on AD pathogenesis and demonstrated that mCRP directly causes cellular impairment in the neurons of AD patients in an ApoE genotype-dependent fashion. This research aimed to uncover the roles of both mCRP and ApoE4 in the development of AD characteristics during chronic inflammation. Even though ApoE4 is considered a major genetic risk factor for AD, the mechanism through which ApoE4 contributes to AD is not fully understood, and future research directions should focus on personalized targeted therapeutic approaches.

Vascular Components of GVU

ECs present a variety of characteristics that guarantee BBB integrity, such as increased expression of tight junction molecules, reduced transcytosis/pinocytosis, and free transport of molecules weighing less than 400kDa. Dysfunction of ECs plays a major role in the pathogenesis of AD by augmenting inflammation, oxidative stress, and contributing to reduced Aβ clearance, impaired CBF, and BBB disruption [15].

Vascular smooth muscle cells (vSMCs) and pericytes are localized in the basement membrane and provide vascular stability, structural support for vessels, and coordinate vasoconstriction and vasodilation. The post-mortem brain tissue of AD patients showed loss or/and injury of pericytes. Affected pericytes present mitochondrial abnormalities, intracellular inclusions, pinocytic vesicles, or/and large lipid granules. These microstructural alterations are associated with dilation of vessels, capillary reductions, and a tortuous appearance of vessels [16].

Neuroinflammation: a link between AD progression and GVU

A further hallmark that plays a significant role in the pathogenesis of AD is neuroinflammation - the product of an immune response to Aβ accumulations, displaying both positive and negative effects. During the early onset of the disease, the mechanism responsible for amyloid plaque and reactive oxygen species (ROS) clearance remains efficient; however, prolonged, continuous oxidative stress ultimately affects the immune system by the chronic overproduction of pro-inflammatory molecules and upregulation of the inflammatory process [17].

Cytokines Involved in Neuroinflammation

There is evidence that demonstrates the interrelationship between inflammation and pathogenic events like Aβ accumulation, cognitive deficits, or neuronal loss leading to AD progression [17]. The evidence based on human postmortem brain tissues and amyloid precursor protein (APP) transgenic animals’ brains reveals high levels of interferon gamma (IFN-γ), TNF-α, interleukin-1β (IL-1β). Moreover, interleukin-6 (IL-6) is produced by stimulated astrocytes and microglia in different brain areas, especially around the Aβ plaques [18].

C-reactive protein

Chronic inflammatory status with elevated CRP and mCRP levels may be considered as a cause of neuroinflammation or as a factor providing continuous insult. Hsuchou H et al. [19] demonstrated that high CRP levels can induce reactive gliosis and increased paracellular BBB permeability. CRP undergoes lysophosphocholine (LPC)-phospholipase-C-dependent dissociation to its biologically more active subunit, mCRP, in response to dysfunctional tissue or the presence of activated immune cell membranes [20].

mCRP as a Novel Biomarker of Neuroinflammation

**Figure 2:**
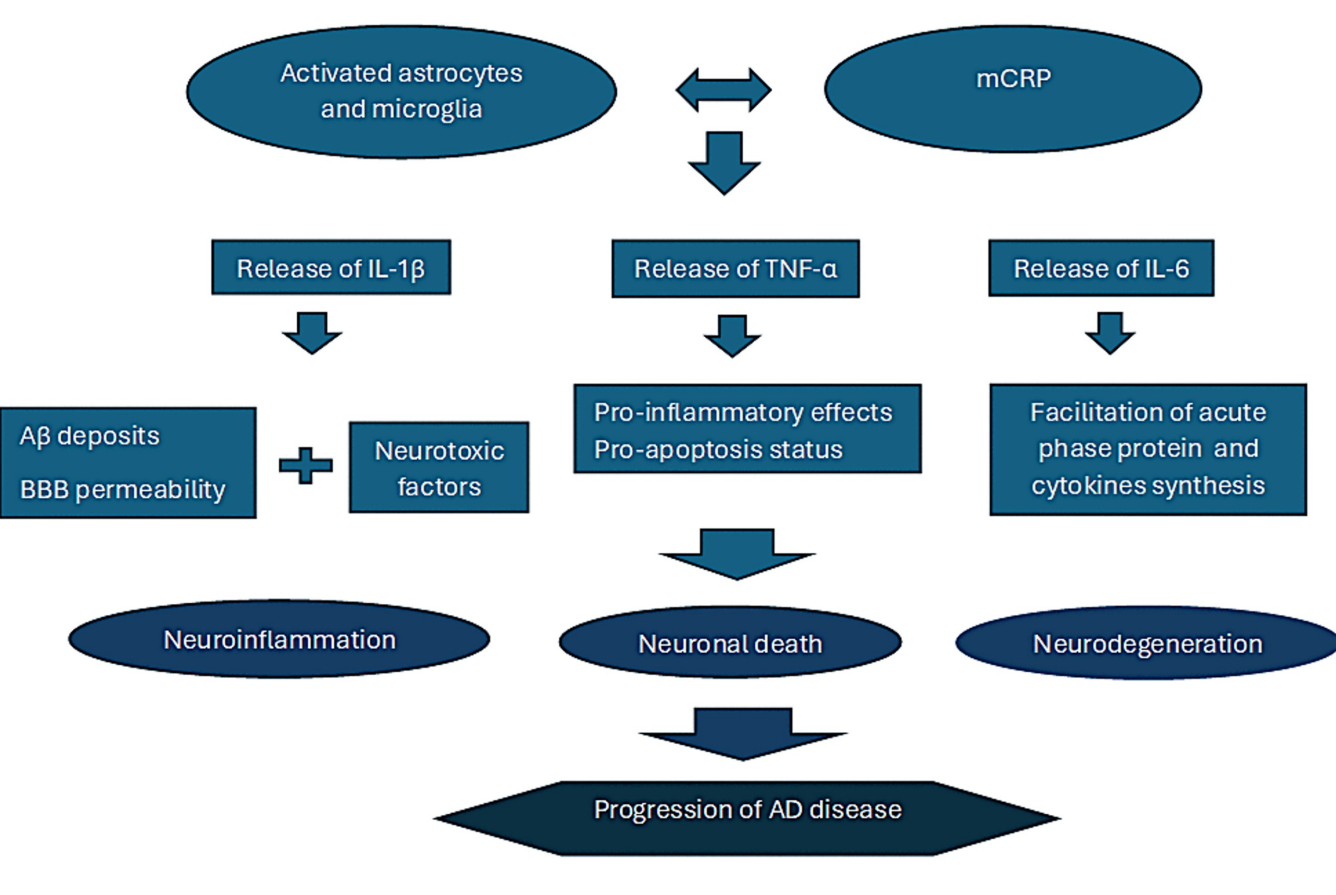
Correlation between mCRP and glia in AD progression. Activation of glial cells, conditioned by inflammatory status and the presence of mCRP, leads to the release of pro-inflammatory cytokines with further negative effects on cerebral blood vessels and neurons (increased BBB permeability, neurotoxic environment, impaired Aβ clearance). A perpetual neuroinflammatory status causes neurodegeneration and progression of AD. mCRP: monomeric C reactive protein; IL-1β: Interleukin-1β; TNF-α: Tumour necrosis factor-α; IL-6: Interleukin-6; AD: Alzheimer's disease; Aβ: Amyloid β. Image Credit: Straistă Mihaela.

The irreversible conversion of CRP to mCRP generates an immediate response, causing local production of monocyte chemoattractant protein-1 (MCP-1), IL-8, TNF-α, IL-6, and associated transcription factors (e.g., NF-κB), promoting a pro-inflammatory micro-environment. Studies indicate that the deposition of mCRP may serve as an early pathological marker and cause of NVU damage, directly connecting neuroinflammation from any source to an increased risk of dementia (Figure 2) [21].

mCRP as a Predictor of Vascular Dysfunction

Li HY et al. [22] showed that mCRP can generate pro-inflammatory EC activation by binding mainly to the apical surface of the blood vessel. This highlights the crucial role of microvessel EC activation in mediating the neuro-inflammatory response following brain injury. mCRP was also found to be responsible for producing leaky and immature vessels with elevated permeability. Novel studies have correlated vascular inflammation with the neurological and cognitive decline seen in AD. In this context, Zhang Z et al. [23] discovered that intraperitoneal injection of mCRP into ApoE4 knock-in mice led to abnormal vascular development and increased T lymphocyte extravasation through a CD31-dependent mechanism, resulting in greater cognitive deficits.

mCRP Extravasation and Distribution in CNS

Thiele JR et al. [24] confirmed that mCRP can induce EC-leukocyte interaction, recruiting them at the site of rat kidney ischemia-reperfusion damage. This finding was later confirmed using the immunofluorescence (IF) technique of biopsied human tissue that underwent renal ischemia/reperfusion injury. Research exploring the mechanism of mCRP stimulation of immune cell activation showed that EC-monocyte adhesion, conditioned by the presence of mCRP, occurred in a fibronectin-dependent profile, representing a significant step in extravasation and upregulation of inflammatory status [25].

**Figure 3:**
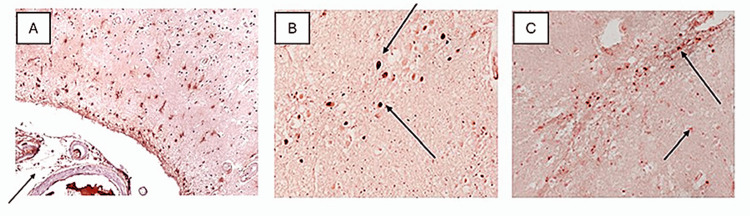
mCRP distribution in IHC study. Figure 3A shows saturated blood vessels (black arrow) with mCRP and cortical infiltration that is stronger in the proximity of vessels (x100). Figures 3B and 3C show strong staining for co-localized mCRP (DAB brown) and CD-68 (nova red) (black arrows) in cells that morphologically appear to be glial cells (x200) (taken from article cited as [26] and used with permission of the publisher). IHC: Immunohistochemistry; mCRP: Monomeric C-reactive protein.

Understanding how mCRP is distributed throughout the brain parenchyma remains challenging. It may diffuse through extracellular spaces, enter the basement membranes of capillaries and arteries, or be synthesized de novo through an unknown pathophysiological response [20]. Immunohistochemical (IHC) studies demonstrated that mCRP staining is present mainly around abnormal-looking areas of brain tissue, with histological evidence of vascular impairment and local inflammation, supporting the hypothesis that affected vessels could release mCRP into the brain parenchyma and instigate local neuroinflammation (Figure 3A). The double labeling with mCRP and CD-68 antibodies showed that there is strong co-localization in glia and associated vessels within damaged areas (Figures 3B-3C) [26].

Novel therapeutic approach regarding mCRP blocking

**Figure 4:**
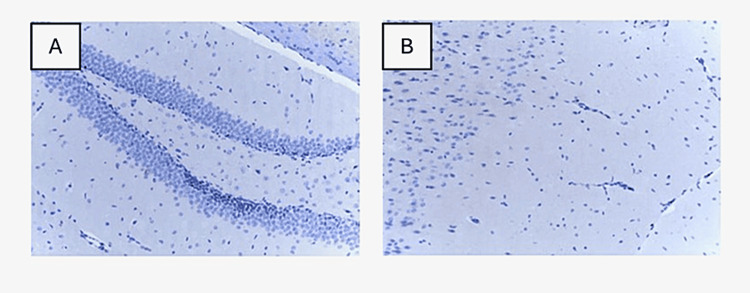
IHC of mCRP and C10M co-injected ApoE -/- mouse. Figure 4A displays negative mCRP staining in the hippocampus (x100). Figure 4B shows no positive staining for mCRP in the cerebral cortex (x100) (taken from the article cited as [27] and used with permission of the publisher). IHC: Immunohistochemistry; mCRP: Monomeric C-reactive protein; ApoE: Apolipoprotein E.

The C10M small molecule is claimed to be a competent dissociation inhibitor. Pastorello Y et al. [27] showed that co-administration of mCRP and C10M in ApoE -/- murine models is efficient in blocking the accumulation of mCRP with negative staining result in IHC study (Figure 4), but further studies are required for a complete understanding.

## Conclusions

AD is a progressive disorder with a complex pathogenesis that involves GVU dysfunction, mCRP involvement, and other biological processes leading to progressive neuronal degeneration and neuroinflammation. The link between the GVU and mCRP is the synergistic inflammatory status generated by cytokines that can compromise BBB integrity, facilitating the entry of peripheral cells and molecules into the brain. The result is chronic and sustained inflammation with progressive neuronal damage and cognitive decline that are typical of AD. Understanding these mechanisms and their interactions can lead to finding potential therapeutic strategies that target mCRP and protect the GVU.
